# 

**Published:** 1858-01

**Authors:** 


PUBLISHED MONTHLY.

VOL. I.] [No. 1.

### THE CHICAGO MEDICAL JOURNAL.

EDITED BY

N. S. DAVIS, M. D.,

AND

W. H. BYFORD, M. D.

JANUARY, 1858.

CHICAGO:

BARNET & CLARKE, BOOK AND JOB PRINTERS, "CHRONOTYPE" OFFICE,

189 LAKE STREET, CORNER OF WELLS.

AT $2.00 PER ANNUM, IN ADVANCE.

